# Rising mortality due to coexisting liver cirrhosis and kidney failure in the United States (1999–2023): A nationwide retrospective analysis

**DOI:** 10.1097/MD.0000000000047662

**Published:** 2026-02-28

**Authors:** Muhammad Shaheer Bin Faheem, Syed Tawassul Hassan, Syeda Umbreen Munir, Muhammad Idrees Khan

**Affiliations:** a Department of Medicine and Surgery, Karachi Institute of Medical Sciences, KIMS, Karachi, Sindh, Pakistan; b Department of Medicine, Karachi Medical and Dental College, KMDC, Karachi, Sindh, Pakistan; c Department of Medicine, Hamdard University, Karachi, Sindh, Pakistan; d Department of Medicine, Adam University School of Medicine, Bishkek, Chuy Region, Kyrgyzstan.

**Keywords:** age adjusted mortality rates, CDC WONDER, gender and racial disparities, kidney failure, liver cirrhosis, mortality

## Abstract

Liver cirrhosis and kidney failure are significant public health concerns, with their coexistence worsening patient outcomes. This study analyzes mortality trends from 1999 to 2023 using Centers for Disease Control and Prevention Wide-Ranging Online Data for Epidemiologic Research data to identify high-risk populations and inform public health strategies. Trends in liver cirrhosis and kidney failure mortality were analyzed using a retrospective analysis of death certificates from the Centers for Disease Control and Prevention Wide-Ranging Online Data for Epidemiologic Research database. Crude and age-adjusted mortality rates (AAMR) per 1,00,000 people and annual percent change in AAMR with 95% confidence interval were obtained and measured across different demographic and geographic subgroups. Liver cirrhosis and kidney failure registered with total 1,36,947 deaths. The AAMR got doubled from 3.6 in 1999 to 6.3 in 2023. No significant variances were appeared from 1999 to 2018 after which a great surge was seen in AAMR till 2023 (annual percent change: 9.63). AAMR remained higher in males as compared to females during the whole study (overall AAMR: Males 5.6 vs Females 3.2) and peaked in non-Hispanic American Indians or Alaska Natives (7.5) among race/ethnicity. The highest crude mortality rate of 8.0 was noted in population aged 75 to 84 years. The overall AAMR of 4.1 remained similar in both metropolitan and nonmetropolitan areas and the South region had the top AAMR of 4.8 considering geographics. Proper resource distribution and more focused approaches are required to tackle the rising shift in mortality rates among different geographics and demographics.

## 1. Introduction

Liver cirrhosis is a leading cause of death, causing 1.47–1.48 million deaths worldwide in 2019.^[1]^ In the United States (US), liver cirrhosis mortality rates have increased over 2 decades (1999: 9.8 vs 2023: 12.2).^[2]^ Further, chronic kidney disease (CKD) is prevalent among 14% to 15% of the adults in the United States, while over 8,08,000 individuals are living with end-stage renal disease (ESRD). Overall, the prevalence of both these conditions has increased by 119% between 1999 and 2019.^[3-5]^ Both CKD and ESRD are the leading causes of mortality and disability and pose a significant cost burden on healthcare, which is estimated at over $1,20,000 per patient annually.^[5,6]^

The coexistence of liver cirrhosis and renal failure can be explained by the overlap of prognostic consequences of hepatic and renal failure. A bidirectional relation has been identified, as liver cirrhosis can lead to renal hypoperfusion and structural kidney failure through its significant effects on the circulatory and hemodynamic system, which includes systemic vasodilation, low effective arterial blood volume and neurohormonal activation.^[7,8]^ Contrastingly, CKD causes systemic inflammation, ammonia clearance impairment and direct tissue damage, all of which can lead to hepatic injury.^[9]^ However, when both conditions co-exist, they synergistically exacerbate metabolic derangements, fluid overload, and multi-organ dysfunction, leading to high hospitalization rates, sepsis, and mortality, with short-term mortality often exceeding 50% to 70%.^[10]^

Improvement is observed in either liver or kidney disease outcomes with the developments in medical therapy and transplantation. For instance, survival outcomes associated with liver cirrhosis have significantly improved with the use of antiviral agents and improvement in portal hypertension management, while outcomes related to advanced CKD/ESRD have shown significant improvements with renal replacement therapy, primarily with transplantation.^[11,12]^ Combined liver-kidney transplantation has also emerged as a life-saving option for patients with concurrent end-stage liver and kidney disease. It provides long-term graft survival and other survival benefits, especially when renal dysfunction is irreversible.^[13]^

However, we still lack long-term national-level mortality data for individuals having both liver cirrhosis and kidney failure. The current literature is limited to the findings from tertiary care or transplant centers, which include highly selective cohorts, and the gap remains in terms of long-term liver cirrhosis and kidney failure-associated mortality patterns, incorporating a national-level sample size. To address this gap, we aimed to examine temporal trends in liver cirrhosis and kidney failure-related mortality in the United States from 1999 to 2023, using data from the Centers for Disease Control and Prevention’s Wide-ranging Online Data for Epidemiologic Research (CDC WONDER) database. We provided a comprehensive overview of the evolving mortality burden related to these coexisting conditions across different demographics and geographics in the United States This will help clinicians to assess the effectiveness of current interventions, guide healthcare policy, and improve outcomes among populations with a higher death burden.

## 2. Materials and Methods

### 2.1. Data source

The CDC WONDER is a publicly available database that stores death certificate information from the National Center for Health Statistics, including underlying and contributing causes of mortality, and covers all 50 states of the United States and the District of Columbia.^[14]^ Further, it provides mortality counts and population-based rates stratified by demographic and geographic factors. In this study, we analyzed mortality trends of the records listing both liver cirrhosis and kidney failure, either as underlying or contributing causes of deaths, from 1999 to 2023, considering the population aged ≥45 years. Identification of relevant deaths was performed using the International Classification of Diseases, Tenth Revision (ICD-10) codes K74.3-K74.6 for liver cirrhosis and N17-N19 for renal failure. Further, the study followed the STROBE guidelines and was exempted from review board approval as it contains de-identified datasets that are made available for public use.

### 2.2. Data extraction

Datasets were retrieved and stratified according to the population size, sex, race/ethnicity, age, location, and geographic details. The age filter (≥45 years) was uniformly applied across all the stratifications, adhering to the predefined inclusion criteria, and ages were divided into 10-year intervals (45–54, 55–64, 65–74, 75–84, and 85+ years) for age-specific analysis. Individuals under the age of 45 were excluded from the analysis due to non-frequent deaths related to coexisting liver cirrhosis and kidney failure in younger demographics. Further, this exclusion allowed us to focus on a more homogenous and clinically relevant cohort in which morbidities are more established, minimizing the statistical noise that might obscure the overall patterns. Racial and ethnic classifications included: non-Hispanic (NH) White, NH Black, NH American Indian/Alaska Native, NH Asian/Pacific Islander, and Hispanic. Medical facilities, hospice care, nursing/long-term care, and the decedent’s home were the reported locations of death. Geographic data were structured using the National Center for Health Statistics’ Urban–Rural Classification Scheme, distinguishing metropolitan and nonmetropolitan areas. The dataset was divided into 4 regions: Northeast, Midwest, South, and West.^[15]^

### 2.3. Statistical analysis

Mortality trends were analyzed by calculating crude and age-adjusted mortality rates (AAMR) per 1,00,000 individuals. The crude mortality rate (CMR) was calculated as the yearly deaths divided by the corresponding US population.^[16]^ AAMRs were standardized to the US population. Trend analysis used the Joinpoint Regression Program (Joinpoint V 5.0, National Cancer Institute, Bethesda) to estimate annual percent change (APC) with 95% confidence intervals (CIs).^[17]^ The program relates longitudinal linear regression to assess significant shifts in mortality trends. It visualizes the changes in trends by incorporating the joinpoints incrementally. Moreover, a logarithmic scale is utilized by this software to represent the increases or decreases in AAMR trends through a slope that notably differs from 0. A method based on a data-driven weighted Bayesian information criterion was employed for model selection, permitting up to 4 joinpoints. Statistical significance was assessed using 2-tailed *t*-tests, with a *P*-value cutoff of .05.

## 3. Result

### 3.1. Proportional mortality rate across different variables from 1999 to 2023

**Figure 1.**
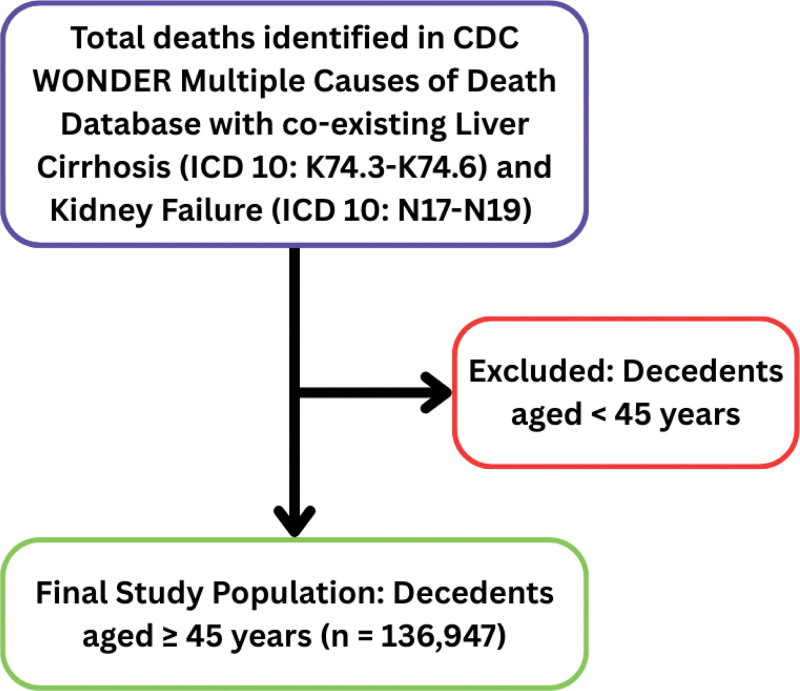
Flowchart of study population selection. Data were extracted from the Centers for Disease Control and Prevention Wide-Ranging Online Data for Epidemiologic Research (CDC WONDER) system. No data counts were suppressed at the national aggregate level for the final study population. CDC WONDER = Centers for Disease Control and Prevention Wide-Ranging Online Data for Epidemiologic Research.

Kidney failure and liver cirrhosis were listed under multiple causes of death in a total of 1,36,947 death certificates of adults aged 45 and above from 1999 to 2023 (Fig. 1). Among all deaths related to these co-occurring conditions, males made up 59%, whereas females accounted for 41% of all deaths. From race/ethnicity, NH whites underwent the highest deaths, making 83% of the total deaths, followed by NH Blacks or African Americans (12%), NH Asians or Pacific Islanders (3%), and NH American Indian or Alaskan Native (2%). In contrast, Hispanics or Latinos accounted for 15% of the overall deaths. The population in the age group 65 to 74 years reported the highest percent total deaths of 29%, followed by the age groups of 55 to 64 (28%), 75 to 84 (21%), 45 to 54 (15%) and lastly the 85+ age group with 7% of the total deaths (Table S1, Supplemental Digital Content, https://links.lww.com/MD/R375)).

Moreover, 83% of all deaths were reported in Metropolitan areas, while the rest, 17%, occurred in nonmetropolitan areas. From overall deaths, 70% occurred in a medical facility-inpatient, 13% in the decedent’s home, 8% in a nursing home/long-term care and 2% each in Medical Facility – outpatient or ER and others. The highest percent of total mortality, 42%, was observed in the South region, followed by the West (22%), the Midwest (20%), and the lowest, 16%, in the Northeast region (Table S1, Supplemental Digital Content, https://links.lww.com/MD/R375).

### 3.2. Overall age adjusted trends for liver cirrhosis and kidney failure related mortality from 1999 to 2023

**Figure 2.**
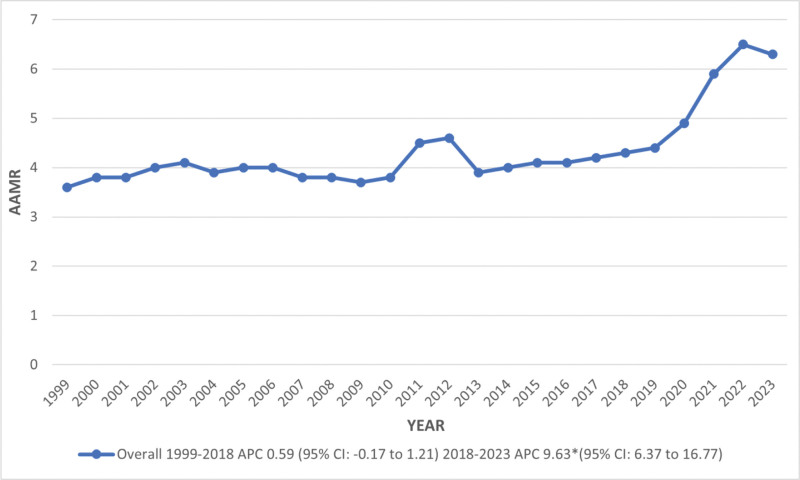
Overall, trends in kidney failure and liver cirrhosis–related age-adjusted mortality rates per 1,00,000 among adults aged 45 and above in the United States, 1999 to 2023. *Indicates that the annual percentage change (APC) is significantly different from 0 at α = 0.05. AAMR **=** age-adjusted mortality rate, APC = annual percentage change, CI = confidence interval.

The overall AAMR almost doubled, rising from 3.6 (95% CI: 3.5–3.7) in 1999 to 6.3 (95% CI: 6.2–6.4) in 2023. However, no significant changes were observed in AAMR from 1999 to 2018, but after that, there was a huge increase in AAMR until 2023 (APC: 9.63; 95% CI: 6.37–16.77; Tables S2 and S3, Supplemental Digital Content, https://links.lww.com/MD/R375; Fig. 2).

### 3.3. Demographic trends

Significant changes in AAMRs were observed among different demographical subgroups in the United States from 1999 to 2023.

#### 3.3.1. Gender stratified

From 1999 to 2023, 81,170 women and 55,777 men died due to kidney failure and liver cirrhosis. However, the overall AAMR in males was almost double that of females (overall AAMR: Males 5.6 vs Females 3.2), and males had continuously higher AAMRs than females throughout the study period (Tables S1 and S2, Supplemental Digital Content, https://links.lww.com/MD/R375).

**Figure 3.**
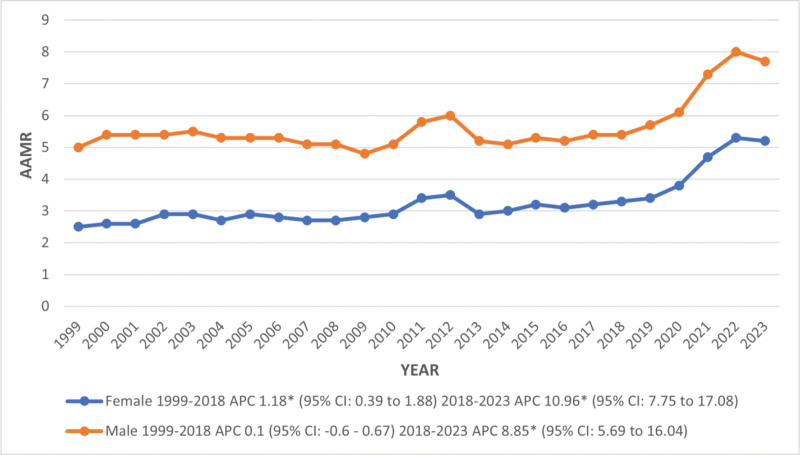
Trends in kidney failure and liver cirrhosis-related age-adjusted mortality rates per 1,00,000, stratified by sex among adults aged 45 and above in the United States, 1999 to 2023. *Indicates that the annual percentage change (APC) is significantly different from 0 at α = 0.05. AAMR **=** age-adjusted mortality rate, APC = annual percentage change, CI = confidence interval.

From the start of the study period till 2018, the AAMR remained stable in males, maintaining a steady increase; in contrast, females showed a significant increase with an associated APC of 1.18 (95% CI: 0.39–1.88). After 2018, the AAMR of both males and females rose sharply till the end of the study period, with an associated APC of 8.85 (95% CI: 5.69–16.04) in males and 10.96 in females (95% CI: 7.75–17.08; Table S3, Supplemental Digital Content, https://links.lww.com/MD/R375; Fig. 3).

#### 3.3.2. Race/ethnicity stratified

Among race/ethnicity, the highest mortality was observed in NH Whites, reporting a total of 1,14,038 deaths, followed by Hispanics or Latinos (deaths: 20,288), NH Black or African American (deaths: 16,503), NH Asian or Pacific Islander (deaths: 4156) and lastly NH American Indian or Alaskan Native with the lowest of total 2062 deaths (Table S1, Supplemental Digital Content, https://links.lww.com/MD/R375).

Conversely, from 1999 to 2023, the peak overall AAMR of 7.5 (95% CI: 5.9–9.5) was observed in NH American Indians or Alaska Natives, followed by 7.4 (95% CI: 6.9–8) in Hispanics or Latinos, 4.9 (95% CI: 4.5–5.3) in NH Blacks or African Americans, 4.3 (95% CI: 4.2–4.4) in NH Whites and least reported in NH Asians or Pacific Islanders 3.2 (95% CI: 2.7–3.7; Table S4, Supplemental Digital Content, https://links.lww.com/MD/R375).

**Figure 4.**
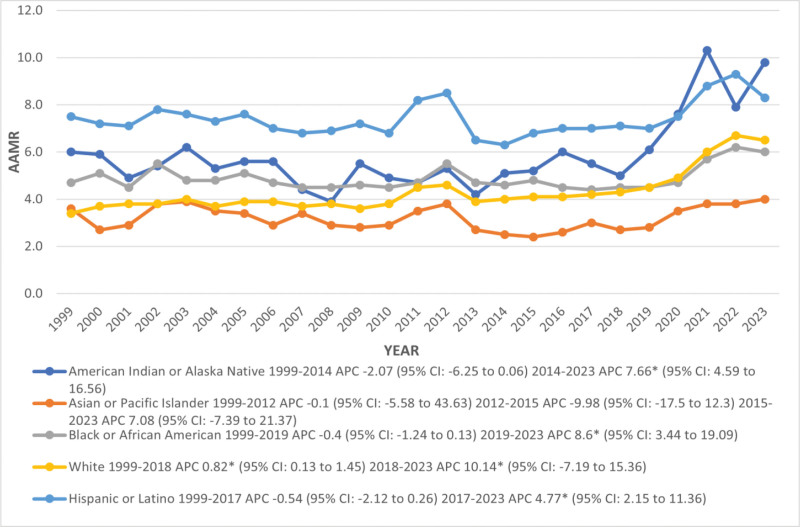
Trends in kidney failure and liver cirrhosis-related age-adjusted mortality rates per 1,00,000, stratified by race and ethnicity among adults aged 45 and above in the United States, 1999 to 2023. AAMR **=** age-adjusted mortality rate, APC = annual percentage change, CI = confidence interval.

From 1999 to 2023, the trend in CMRs increased sharply among all races and ethnicities except in Asians or Pacific Islanders, in which it maintained a steady inclined state with no significant changes in trends (1999–2023 AAPC: NH White: 2.70; 95% CI: 2.30–3.17, NH American Indians or Alaska Natives: 1.5; 95% CI: 0.45–2.67, NH Blacks or African Americans: 1.05; 95% CI: 0.46–1.59 and Hispanics or Latinos: 0.76; 95% CI: 0.14–1.39; Table S5, Supplemental Digital Content, https://links.lww.com/MD/R375; Fig. 4).

#### 3.3.3. Age group stratified

Most of the deaths occurred in adults aged 65 to 74 years (deaths: 40,384) and 55 to 64 years with 37,687 deaths, followed by the individuals aged 75 to 84 years with a total of 28,676 deaths, 45 to 54 years with a total of 20,966 deaths and the age group of 85+ years reported the lowest deaths (9234 deaths). Adults between the age group 75 to 84 years had the highest average CMR of 8.0 (95% CI: 7.5–8.5) followed by those aged 85+ years 6.5 (95% CI: 5.8–7.1), 65 to 74 years 6.3 (95% CI: 6.0–6.7), 55 to 64 years 4.1 (95% CI: 4.0–4.3) and minimum of 2.0 (95% CI: 1.9–2.1) recorded in those aged between 45 and 54 years (Tables S1 and S6, Supplemental Digital Content, https://links.lww.com/MD/R375).

The CMR significantly heightened from 1999 across individuals under age groups of 75 to 84 years and 85+ years till 2018 with associated APCs of 1.18 and 1.85, respectively and increased up to 2019 for those aged 55 to 64 years with APCs of 1.23 (95% CI: 0.53–1.73), after maintaining the increase in trends, the CMR made a far more significant spike till the end of study duration (2018–2023: 75–84 years APC: 10.57; 95% CI: 6.36–21.4; 85+ years APC: 12.65; 95% CI: 8.81–20.16 and 2019–2023: 55–64 years APC: 7.41; 95% CI: 3.75–15.34; Table S3, Supplemental Digital Content, https://links.lww.com/MD/R375; Fig. 4).

No significant changes in CMR were observed in the initial years among the individuals aged 45 to 54 and 65 to 74 years. However, the CMR considerably declined from 2001 to 2018 in individuals aged 45 to 54 years (APC: −1.98; −9.13 to −1.35) and then rose sharply up till 2023 (2018–2023 APC: 9.7; 5.1–20.69). After the period of stability till 2014 in adults aged 65 to 74 years, the CMR took rise till 2019, after which a significant increase in CMR values was seen before 2023 (2014–2019 APC: 3.77; 95% CI: 0.56–7.48 and 2019–2023 APC: 11.7; 95% CI: 8.41–19.09; Table S3, Supplemental Digital Content, https://links.lww.com/MD/R375; Fig. S1, Supplemental Digital Content, https://links.lww.com/MD/R375).

### 3.4. Geographic trends

Significant variations were observed in mortality rates across different geographical classifications in the US throughout our study.

#### 3.4.1. Urbanization

**Figure 5.**
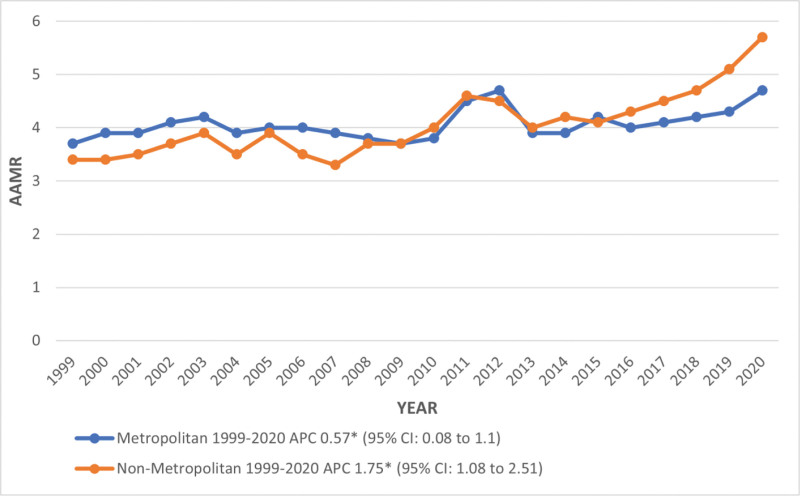
Trends in kidney failure and liver cirrhosis-related age-adjusted mortality rates per 1,00,000, stratified by urbanization among adults aged 45 and above in the United States, 1999 to 2020. *Indicates that the annual percentage change (APC) is significantly different from 0 at α = 0.05. AAMR **=** age-adjusted mortality rate, APC = annual percentage change, CI = confidence interval.

Metropolitan areas reported higher deaths than nonmetropolitan areas (deaths: 89,912 vs 18,601), while the overall AAMR of 4.1 was the same for both metropolitan and nonmetropolitan areas. Similarly, the trends in AAMR significantly increased in both areas from 1999 to 2020 (metropolitan APC: 0.57; 95% CI: 0.08–1.1 and nonmetropolitan APC: 1.75; 1.08–2.51; Tables S1, S3, and S7, Supplemental Digital Content, https://links.lww.com/MD/R375; Fig. 5).

#### 3.5.2. Census regions

The region of the South had the highest mortality, accounting for a total of 57,159 deaths, followed by the regions of the West and Midwest (deaths: 30,507 and 27,359), while the lowest deaths were reported from the Northeast region (deaths: 21,922). Significant changes were seen in AAMRs among different regions, in which the region of South reported the highest overall AAMR of 4.8 (95% CI: 4.6–5), followed by the west and mid-west regions with AAMRs of 4.4 (95% CI: 4.1–4.6) and 4.0 (95% CI: 3.7–4.2). In comparison, the lowest AAMR of 3.7 (95% CI: 3.5–4) was observed in the northeast region (Tables S1 and S8, Supplemental Digital Content, https://links.lww.com/MD/R375).

From 1999 to 2018, the AAMRs showed a significant incline in the south region with an associated APC of 1.42 (95% CI: 0.52–2.03), whereas they declined in the northeast regions with an associated APC of −0.75 (95% CI: −1.54 to −0.11) and remained stable in the regions of the Midwest and West. Then from 2018 till 2023 the AAMRs were considerably raised among all regions (2018–2023: Midwest APC: 11.24; 95% CI: 7.58–20.34, West APC: 10.64; 95% CI: 6.01 to 23.05, Northeast APC: 9.53; 95% CI: 5. –16.97 and South APC: 8.22; 95% CI: 5.13–15.8; Table S3, Supplemental Digital Content, https://links.lww.com/MD/R375; Fig. S2, Supplemental Digital Content, https://links.lww.com/MD/R375).

#### 3.5.3. Place of deaths

About 95,903 deaths were recorded in inpatient medical facilities by the end of the study, followed by 18,357 and 10,595 deaths in Decedents and nursing homes. The last deaths were reported from outpatient or ER facilities and others (deaths: 2483 and 2656; Table S1, Supplemental Digital Content, https://links.lww.com/MD/R375; Fig. S3, Supplemental Digital Content, https://links.lww.com/MD/R375).

#### 3.6.4. States

Significant disparities were noted across all 50 states and the District of Columbia, with the state of Texas representing the highest AAMR of 7.6 (95% CI: 7.4–7.7) and the state of Wyoming with the least AAMR of 2.2 (95% CI: 1.7–2.6). The average AAMR of the states: New Mexico (5.2), Kentucky (5.3), Rhode Island (5.4), West Virginia (5.9) and Texas (7.6) that were included in top 90th percentile is 2.3 times greater than the AAMRs of the states: Wyoming (2.2), Nebraska (2.5), Iowa (2.6), Louisiana (2.6), Montana (2.7) and New York (2.7) underlaid lower 10th percentile (Table S9, Supplemental Digital Content, https://links.lww.com/MD/R375; Fig. S4, Supplemental Digital Content, https://links.lww.com/MD/R375).

## 4. Discussion

In our 25-year analysis of mortality data from the Centers for Disease Control and Prevention, we report significant trends. First, overall mortality concerning AAMR increased from 1999 to 2023, with more pronounced mortality trends seen in males as compared to females. Second, racial trends reported the highest death rates in NH Whites. Third, we can see a comparable and higher mortality rate in age groups 65 to 74 and 55 to 64. Among census regions, the South had the highest number of deaths due to kidney failure and liver cirrhosis, and a large proportion of deaths happened in metropolitan areas as compared to nonmetropolitan areas. Lastly, the majority of deaths occurred in inpatient medical facilities rather than in other locations.

Our study showed increased mortality trends in patients with liver cirrhosis complicated with kidney failure, which was also apparent from many previous studies. One of the studies conducted in 2012 by Fede et al also demonstrated an increase in mortality in cirrhosis with renal failure and highlighted that renal failure increases the chances of mortality by 7-fold, and 50% of patients died within 1 month. The reason for poor prognosis after the occurrence of renal failure is not known, but many advocate that renal failure is simply a marker of advanced liver disease. Therefore, efforts should focus on preventing renal failure by addressing its precipitating factors and ensuring early diagnosis. Studies have shown that nonselective beta-blockers, when effectively reducing hepatic venous pressure gradient, can lower the incidence of hepatorenal syndrome (HRS).

Additionally, prophylactic antibiotic therapy in patients with advanced cirrhosis or those at risk of spontaneous bacterial peritonitis has been found to reduce the long-term risk of renal failure.^[18]^ Similarly, a 2016 study conducted in Taiwan found that ESRD significantly heightened the 3-year mortality risk in cirrhosis patients, doubling the likelihood of death, particularly in those with recent complications.^[19]^ Additionally, research by Duah et al in Ghana revealed a high mortality rate among patients with liver cirrhosis attributed to acute kidney injury (AKI).^[20]^

The incidence of acute renal failure (ARF) was more commonly observed in males in both viral and alcohol-induced liver cirrhosis, and a higher mortality rate due to ARF, which is due to the presence of hyponatremia, hyperbilirubinemia, high serum liver enzyme concentrations, infections (especially septicemia), and GI bleeding.^[21]^ Similarly, A 2006 study showed that renal disorders in liver cirrhosis were most frequent, and these conditions were more common in males and significantly contributed to higher mortality rates in advanced cirrhosis, with the presence of renal disease further exacerbating the risk of death.^[22]^ Moreover, a study done by Mazumder et al in 2020 revealed that the female sex had a decreased risk of all-cause mortality as compared to their male counterparts because they tend to have fewer complications of cirrhosis, lower MELD-Na score and lower rates of hepatocellular carcinoma, but the liver-related mortality remained the same among both genders.^[23]^ This aligns with our study’s findings, which indicate a higher mortality trend among male patients with liver cirrhosis caused by kidney failure.

A prospective study conducted in India in 2024 revealed a high prevalence of and mortality due to AKI in cirrhosis patients, with a mean age of 51.46. Another study showed a higher likelihood of AKI in patients aged 60 to 69 years, followed by those in the 40 to 49 age group.^[24,25]^ Increased direct bilirubin levels and higher MELD scores were strongly linked to the development of AKI. Additionally, AKI and MELD scores were recognized as independent predictors of mortality at 30 and 90 days. Survival outcomes varied based on the type and severity of AKI, with patients experiencing AKI stage 3 and acute tubular necrosis facing significantly higher mortality rates. Timely detection and effective management of AKI are essential in reducing mortality risk among patients with liver cirrhosis.^[24]^ Whereas a study by Carvalho et al reported higher in-hospital mortality due to renal failure, particularly among patients with a mean age of 66. Renal failure is prevalent in nearly half of individuals with decompensated end-stage liver disease, primarily triggered by hypovolemia and bacterial infections. Its occurrence significantly impacts survival outcomes, especially in patients with bacterial infections and type 1 hepatorenal syndrome (HRS), who remain at high risk for progressive renal dysfunction despite intensive medical intervention,^[25]^ which aligns to some extent with our findings.

Furthermore, our analysis showed higher mortality trends in NH Whites. In contrast, a 2021 study found that Black patients had the highest rates of hypertension, diabetes and CKD and suffered more all-cause mortality and non-liver-related deaths in Cirrhosis patients and were less likely to be listed or transplanted.^[26]^ Utilizing National Inpatient Sample (NIS) database from 2009 to 2018, a study found that Black patients suffering from decompensated cirrhosis are more likely to receive hemodialysis for AKI and HRS and have a higher chance of death and less likely to receive transplant as compared to Whites because liver transplantation involves multiple challenges, such as insurance approval, social support, sobriety, and medical clearance, hemodialysis is more accessible and Medicaid and Medicare cover it and has fewer contraindications compared to liver transplantation.^[27]^

A 2020 study utilizing the National Inpatient Sample (NIS) database from 2004 to 2016 identified notable disparities in AKI prevalence among cirrhosis patients, with higher rates observed in teaching hospitals compared to rural facilities. While cirrhosis cases were most prevalent in the South, the West showed a slightly higher burden of AKI. The study also emphasized that AKI significantly increases mortality, particularly in cirrhosis patients with infections and portal hypertension-related complications,^[28]^ potentially due to disparities in healthcare costs and access. These findings closely align with our study.

A study done in 2011 by Warner et al found the highest mortality trends in hospitalized patients with cirrhosis due to acute kidney Iinjury (AKI), especially in patients with type 1 hepatorenal syndrome (HRS).^[29]^ Likewise, a 2021 study by Cullaro et al, utilizing the National Inpatient Sample (NIS), identified a significant burden of kidney dysfunction, particularly acute kidney injury (AKI), among hospitalized patients with liver cirrhosis. The study further highlighted that patients with AKI had an elevated risk of in-hospital mortality. At the same time, those with both AKI and CKD faced an even greater risk of death compared to those with CKD alone.^[30]^ This finding is similar to our analysis that the majority of deaths happened in inpatient facilities as compared to other locations.

## 5. Study limitations

Our study has several limitations. First, as a retrospective analysis utilizing mortality data from the Centers for Disease Control and Prevention (CDC), it is prone to potential inaccuracies in cause-of-death reporting and coding errors. Second, the dataset lacks detailed clinical information, including laboratory findings, severity scores such as MELD-Na, and specific treatment strategies, which could offer deeper insights into patient outcomes. Third, the absence of individual-level data limits our ability to assess key factors such as socioeconomic status, coexisting medical conditions, and access to specialized healthcare. Additionally, our study does not distinguish between different causes of liver cirrhosis, such as alcohol-related cirrhosis, viral hepatitis, or NAFLD, all of which could impact mortality trends. Lastly, while our analysis highlights important associations between AKI, cirrhosis, and mortality, its observational design does not allow for establishing causation. Future research incorporating prospective cohort studies and clinical data must confirm and further explore these findings.

## 6. Conclusion

In conclusion, our 25-year analysis highlights a rising burden of mortality associated with liver cirrhosis complicated by kidney failure, with significant disparities across gender, race, age groups, and geographic regions. Males, NH Whites, and older adults exhibited the highest mortality trends, with the South experiencing the most significant disease burden. Hospitalized patients, particularly those in metropolitan areas, faced the highest mortality rates, underscoring the critical role of inpatient care. Our findings align with previous studies demonstrating the detrimental impact of renal dysfunction on survival in cirrhotic patients, emphasizing the need for early detection, preventive strategies, and improved access to specialized care to mitigate mortality risks.

## Author contributions

**Conceptualization:** Muhammad Shaheer Bin Faheem, Syed Tawassul Hassan.

**Data curation:** Syed Tawassul Hassan.

**Formal analysis:** Muhammad Shaheer Bin Faheem, Syed Tawassul Hassan.

**Project administration:** Muhammad Shaheer Bin Faheem, Syed Tawassul Hassan, Muhammad Idrees Khan.

**Resources:** Syeda Umbreen Munir, Muhammad Idrees Khan.

**Software:** Syed Tawassul Hassan.

**Supervision:** Muhammad Shaheer Bin Faheem.

**Validation:** Syeda Umbreen Munir.

**Visualization:** Muhammad Shaheer Bin Faheem.

**Writing – original draft:** Muhammad Shaheer Bin Faheem, Syed Tawassul Hassan, Syeda Umbreen Munir.

**Writing – review & editing:** Muhammad Shaheer Bin Faheem, Syed Tawassul Hassan.

## Supplementary Material


