# Supplementary material for: Rising mortality due to coexisting liver cirrhosis and kidney failure in the United States (1999–2023): A nationwide retrospective analysis
Source: Medicine (Baltimore). 2026 Feb 28;105(9):e47662. doi: 10.1097/MD.0000000000047662 (PMC12956238; doi:10.1097/MD.0000000000047662)
Supplement: Supplementary file 1 [file medi-105-e47662-s001.docx]

|  | **Deaths** | **% of Total Deaths** |
| --- | --- | --- |
| Overall | 136947 | 100% |
| **Gender** | | |
| Female | 55777 | 41% |
| Male | 81170 | 59% |
| **Race** | | |
| NH White | 114038 | 83% |
| NH Black or African American | 16503 | 12% |
| NH Asian or Pacific Islander | 4156 | 3% |
| NH American Indian or Alaskan Native | 2062 | 2% |
| Hispanic or Latino | 20288 | 15% |
| **Age Groups** | | |
| 45-54 years | 20966 | 15% |
| 55-64 years | 37687 | 28% |
| 65-74 years | 40384 | 29% |
| 75-84 years | 28676 | 21% |
| 85+ years | 9234 | 7% |
| **Place of Deaths** | | |
| Medical Facility - Inpatient | 95903 | 70% |
| Medical Facility - Outpatient or ER | 2483 | 2% |
| Decedent's home | 18357 | 13% |
| Nursing home/long term care | 10595 | 8% |
| Other | 2656 | 2% |
| **Urbanization** | | |
| Metropolitan Areas | 89912 | 83% |
| Non-Metropolitan Areas | 18601 | 17% |
| **Regions** | | |
| Northeast | 21922 | 16% |
| Midwest | 27359 | 20% |
| South | 57159 | 42% |
| West | 30507 | 22% |
| **NH: Non-Hispanic** | | |

**Supplemental Table 1.** Absolute number of Kidney Failure and Liver Cirrhosis-related deaths and percent total deaths among adults aged 45 and above stratified by overall, sex, race/ ethnicity, age group, place of death, urbanization and regions in the United States, 1999-2023.

| **Age-Adjusted Rate /100,000 (95% CI)** | | | |
| --- | --- | --- | --- |
| **Year** | **Overall** | **Male** | **Female** |
| 1999 | 3.6 (3.5 - 3.7) | 5 (4.8 - 5.2) | 2.5 (2.3 - 2.6) |
| 2000 | 3.8 (3.7 - 3.9) | 5.4 (5.2 - 5.6) | 2.6 (2.5 - 2.7) |
| 2001 | 3.8 (3.7 - 4) | 5.4 (5.2 - 5.6) | 2.6 (2.4 - 2.7) |
| 2002 | 4 (3.9 - 4.1) | 5.4 (5.2 - 5.6) | 2.9 (2.7 - 3) |
| 2003 | 4.1 (3.9 - 4.2) | 5.5 (5.3 - 5.8) | 2.9 (2.8 - 3) |
| 2004 | 3.9 (3.8 - 4) | 5.3 (5 - 5.5) | 2.7 (2.6 - 2.9) |
| 2005 | 4 (3.9 - 4.1) | 5.3 (5.1 - 5.5) | 2.9 (2.8 - 3.1) |
| 2006 | 4 (3.9 - 4.1) | 5.3 (5.1 - 5.5) | 2.8 (2.6 - 2.9) |
| 2007 | 3.8 (3.7 - 3.9) | 5.1 (4.9 - 5.3) | 2.7 (2.6 - 2.8) |
| 2008 | 3.8 (3.7 - 4) | 5.1 (4.9 - 5.3) | 2.7 (2.6 - 2.9) |
| 2009 | 3.7 (3.6 - 3.8) | 4.8 (4.6 - 5) | 2.8 (2.6 - 2.9) |
| 2010 | 3.8 (3.7 - 4) | 5.1 (4.9 - 5.3) | 2.9 (2.7 - 3) |
| 2011 | 4.5 (4.4 - 4.6) | 5.8 (5.6 - 6) | 3.4 (3.2 - 3.5) |
| 2012 | 4.6 (4.5 - 4.8) | 6 (5.8 - 6.3) | 3.5 (3.3 - 3.6) |
| 2013 | 3.9 (3.8 - 4) | 5.2 (5 - 5.4) | 2.9 (2.7 - 3) |
| 2014 | 4 (3.9 - 4.1) | 5.1 (4.9 - 5.3) | 3 (2.9 - 3.1) |
| 2015 | 4.1 (4 - 4.2) | 5.3 (5.1 - 5.5) | 3.2 (3.1 - 3.3) |
| 2016 | 4.1 (4 - 4.2) | 5.2 (5 - 5.4) | 3.1 (3 - 3.3) |
| 2017 | 4.2 (4.1 - 4.3) | 5.4 (5.2 - 5.5) | 3.2 (3 - 3.3) |
| 2018 | 4.3 (4.2 - 4.4) | 5.4 (5.3 - 5.6) | 3.3 (3.2 - 3.5) |
| 2019 | 4.4 (4.3 - 4.6) | 5.7 (5.5 - 5.9) | 3.4 (3.3 - 3.5) |
| 2020 | 4.9 (4.7 - 5) | 6.1 (5.9 - 6.3) | 3.8 (3.7 - 3.9) |
| 2021 | 5.9 (5.8 - 6) | 7.3 (7.1 - 7.5) | 4.7 (4.6 - 4.9) |
| 2022 | 6.5 (6.4 - 6.6) | 8 (7.8 - 8.2) | 5.3 (5.1 - 5.4) |
| 2023 | 6.3 (6.2 - 6.4) | 7.7 (7.5 - 7.9) | 5.2 (5 - 5.3) |
| **Total** | 4.3 (4.2 - 4.4) | 5.6 (5.4 - 5.8) | 3.2 (3.1 - 3.4) |

**Supplemental Table 2.** Overall and sex-stratified Kidney Failure and Liver Cirrhosis-related age-adjusted mortality rates per 100,000 among adults aged 45 and above in the United States, 1999 to 2023.

| **Year Interval** | **APC (95% CI)** |
| --- | --- |
| **Overall** | |
| 1999-2018 | 0.59 (-0.17 - 1.21) |
| 2018-2023 | 9.63* (6.37 - 16.77) |
| **Gender** | |
| **Male** | |
| 1999-2018 | 0.1 (-0.6 - 0.67) |
| 2018-2023 | 8.85* (5.69 - 16.04) |
| **Female** | |
| 1999-2018 | 1.18* (0.39 - 1.88) |
| 2018-2023 | 10.96* (7.75 - 17.08) |
| **Race** | |
| **NH American Indian or Alaska Native** | |
| 1999-2014 | -2.07 (-6.25 - 0.06) |
| 2014-2023 | 7.66* (4.59 - 16.56) |
| **NH Asian or Pacific Islander** | |
| 1999-2012 | -0.1 (-5.58 - 43.63) |
| 2012-2015 | -9.98 (-17.5 - 12.3) |
| 2015-2023 | 7.08 (-7.39 - 21.37) |
| **NH Black or African American** | |
| 1999-2019 | -0.4 (-1.24 - 0.13) |
| 2019-2023 | 8.6* (3.44 - 19.09) |
| **NH White** | |
| 1999-2018 | 0.82* (0.13 - 1.45) |
| 2018-2023 | 10.14* (7.19 - 15.36) |
| **Hispanic or Latino** | |
| 1999-2017 | -0.54 (-2.12- 0.26) |
| 2017-2023 | 4.77* (2.15 - 11.36) |
| **Age Groups** | |
| **45-54 years** | |
| 1999-2001 | 12.49 (-0.81 - 27.04) |
| 2001-2018 | -1.98* (-9.13 - -1.35) |
| 2018-2023 | 9.7* (5.1 - 20.69) |
| **55-64 years** | |
| 1999-2019 | 1.23* (0.53 - 1.73) |
| 2019-2023 | 7.41* (3.75 - 15.34) |
| **65-74 years** | |
| 1999-2014 | -0.08 (-3.56 - 0.77) |
| 2014-2019 | 3.77* (0.56 - 7.48) |
| 2019-2023 | 11.7* (8.41 - 19.09) |
| **75-84 years** | |
| 1999-2018 | 1.18* (0.16 - 1.85) |
| 2018-2023 | 10.57* (6.36 - 21.4) |
| **85+ years** | |
| 1999-2018 | 2* (1.12 - 2.78) |
| 2018-2023 | 12.65* (8.81 - 20.16) |
| **Urbanization** | |
| **Metropolitan** | |
| 1999-2020 | 0.57* (0.08 - 1.1) |
| **Non-Metropolitan** | |
| 1999-2020 | 1.75* (1.08 - 2.51) |
| **Regions** | |
| **Northeast** | |
| 1999-2018 | -0.75* (-1.54 - -0.11) |
| 2018-2023 | 9.53* (5.65 - 16.97) |
| **Midwest** | |
| 1999-2018 | 0.34 (-0.5 - 1.05) |
| 2018-2023 | 11.24* (7.58 - 20.34) |
| **South** | |
| 1999-2018 | 1.42* (0.52 - 2.03) |
| 2018-2023 | 8.22* (5.13 - 15.8) |
| **West** | |
| 1999-2018 | 0.26 (-1.02 - 1.08) |
| 2018-2023 | 10.64* (6.01 - 23.05) |
| **NH: Non-Hispanic; APC: Annual Percent Change** | |

**Supplemental Table 3.** Annual percent change (APC) of Kidney Failure and Liver Cirrhosis–related age-adjusted mortality rates per 100,000 among adults aged 45 and above in the United States, 1999 to 2023.

| **Age-Adjusted Rate /100,000 (95% CI)** | | | | | |
| --- | --- | --- | --- | --- | --- |
| **Year** | **NH American Indian or Alaska Native** | **NH Asian or Pacific Islander** | **NH Black or African American** | **NH White** | **Hispanic or Latino** |
| 1999 | 8.2 (6 - 11.1) | 3.6 (2.9 - 4.5) | 4.7 (4.3 - 5.2) | 3.4 (3.3 - 3.6) | 7.5 (6.8 - 8.3) |
| 2000 | 8.2 (5.9 - 10.9) | 2.7 (2.1 - 3.4) | 5.1 (4.6 - 5.5) | 3.7 (3.6 - 3.8) | 7.2 (6.5 - 8) |
| 2001 | 6.8 (4.9 - 9.3) | 2.9 (2.3 - 3.6) | 4.5 (4.1 - 5) | 3.8 (3.6 - 3.9) | 7.1 (6.5 - 7.8) |
| 2002 | 7.4 (5.4 - 9.8) | 3.8 (3.1 - 4.6) | 5.5 (5.1 - 6) | 3.8 (3.7 - 3.9) | 7.8 (7.1 - 8.5) |
| 2003 | 8.3 (6.2 - 10.9) | 3.9 (3.2 - 4.6) | 4.8 (4.4 - 5.2) | 4 (3.8 - 4.1) | 7.6 (6.9 - 8.2) |
| 2004 | 7.3 (5.3 - 9.8) | 3.5 (2.8 - 4.1) | 4.8 (4.4 - 5.2) | 3.7 (3.6 - 3.9) | 7.3 (6.7 - 8) |
| 2005 | 7.4 (5.6 - 9.7) | 3.4 (2.7 - 4) | 5.1 (4.7 - 5.5) | 3.9 (3.8 - 4) | 7.6 (7 - 8.3) |
| 2006 | 7.4 (5.6 - 9.6) | 2.9 (2.3 - 3.5) | 4.7 (4.3 - 5.1) | 3.9 (3.8 - 4) | 7 (6.4 - 7.6) |
| 2007 | 6.1 (4.4 - 8.1) | 3.4 (2.8 - 4) | 4.5 (4.1 - 4.9) | 3.7 (3.6 - 3.8) | 6.8 (6.2 - 7.3) |
| 2008 | 5.3 (3.9 - 7.1) | 2.9 (2.3 - 3.4) | 4.5 (4.2 - 4.9) | 3.8 (3.6 - 3.9) | 6.9 (6.3 - 7.5) |
| 2009 | 7.3 (5.5 - 9.4) | 2.8 (2.2 - 3.3) | 4.6 (4.2 - 5) | 3.6 (3.5 - 3.7) | 7.2 (6.6 - 7.7) |
| 2010 | 6.5 (4.9 - 8.5) | 2.9 (2.4 - 3.4) | 4.5 (4.1 - 4.9) | 3.8 (3.7 - 3.9) | 6.8 (6.2 - 7.3) |
| 2011 | 6.2 (4.7 - 8.1) | 3.5 (2.9 - 4) | 4.7 (4.3 - 5) | 4.5 (4.4 - 4.6) | 8.2 (7.7 - 8.8) |
| 2012 | 6.9 (5.3 - 8.8) | 3.8 (3.3 - 4.4) | 5.5 (5.1 - 5.9) | 4.6 (4.4 - 4.7) | 8.5 (8 - 9.1) |
| 2013 | 5.5 (4.2 - 7) | 2.7 (2.3 - 3.2) | 4.7 (4.3 - 5.1) | 3.9 (3.7 - 4) | 6.5 (6 - 7) |
| 2014 | 6.5 (5.1 - 8.3) | 2.5 (2.1 - 2.9) | 4.6 (4.3 - 5) | 4 (3.8 - 4.1) | 6.3 (5.8 - 6.7) |
| 2015 | 6.5 (5.2 - 8.1) | 2.4 (2 - 2.8) | 4.8 (4.5 - 5.2) | 4.1 (4 - 4.2) | 6.8 (6.3 - 7.3) |
| 2016 | 7.6 (6 - 9.1) | 2.6 (2.2 - 3) | 4.5 (4.2 - 4.9) | 4.1 (4 - 4.2) | 7 (6.5 - 7.5) |
| 2017 | 6.8 (5.5 - 8.5) | 3 (2.5 - 3.4) | 4.4 (4.1 - 4.8) | 4.2 (4.1 - 4.3) | 7 (6.5 - 7.4) |
| 2018 | 6.3 (5 - 7.9) | 2.7 (2.3 - 3.1) | 4.5 (4.2 - 4.8) | 4.3 (4.2 - 4.4) | 7.1 (6.7 - 7.6) |
| 2019 | 7.6 (6.1 - 9) | 2.8 (2.4 - 3.2) | 4.5 (4.2 - 4.8) | 4.5 (4.4 - 4.7) | 7 (6.6 - 7.5) |
| 2020 | 9.2 (7.6 - 10.8) | 3.5 (3.1 - 3.9) | 4.7 (4.4 - 5.1) | 4.9 (4.8 - 5.1) | 7.5 (7 - 7.9) |
| 2021 | 12.1 (10.3 - 14) | 3.8 (3.3 - 4.2) | 5.7 (5.3 - 6.1) | 6 (5.8 - 6.1) | 8.8 (8.3 - 9.3) |
| 2022 | 9.5 (7.9 - 11.1) | 3.8 (3.4 - 4.3) | 6.2 (5.9 - 6.6) | 6.7 (6.6 - 6.9) | 9.3 (8.8 - 9.8) |
| 2023 | 11.5 (9.8 - 13.3) | 4 (3.6 - 4.4) | 6 (5.7 - 6.4) | 6.5 (6.4 - 6.7) | 8.3 (7.9 - 8.7) |
| **Total** | 7.5 (5.9 - 9.5) | 3.2 (2.7 - 3.7) | 4.9 (4.5 - 5.3) | 4.3 (4.2 - 4.4) | 7.4 (6.9 - 8) |
| **NH: Non-Hispanic** | | | | | |

**Supplemental Table 4.** Race/ Ethnicity stratified Kidney Failure and Liver Cirrhosis-related age-adjusted mortality rates per 100,000 among adults aged 45 and above in the United States, 1999 to 2023.

| **Race** | **AAPC (95% CI)** |
| --- | --- |
| NH American Indian or Alaska Native | 1.47 (0.45 - 2.67) |
| NH Asian or Pacific Islander | 0.92 (-0.43 - 3.82) |
| NH Black or African American | 1.05 (0.46 - 1.59) |
| NH White | 2.7 (2.3 - 3.17) |
| Hispanic or Latino | 0.76 (0.14 - 1.39) |

**Supplemental Table 5.** Trends in Kidney Failure and Liver Cirrhosis-related average annual per cent change (AAPC) stratified by race and ethnicity in the United States, 1999 to 2023.

| **Crude Rate /100,000 (95% CI)** | | | | | |
| --- | --- | --- | --- | --- | --- |
| **Year** | **45-54 years** | **55-64 years** | **65-74 years** | **75-84 years** | **85+ years** |
| 1999 | 1.8 (1.7 - 1.9) | 3.4 (3.2 - 3.6) | 5.7 (5.4 - 6.1) | 6 (5.6 - 6.5) | 4.5 (3.9 - 5.2) |
| 2000 | 2.1 (2 - 2.3) | 3.8 (3.5 - 4) | 5.5 (5.2 - 5.9) | 6.1 (5.7 - 6.6) | 5 (4.3 - 5.6) |
| 2001 | 2.2 (2.1 - 2.4) | 3.5 (3.3 - 3.8) | 5.6 (5.2 - 5.9) | 6.4 (6 - 6.9) | 5.1 (4.5 - 5.8) |
| 2002 | 2.3 (2.2 - 2.5) | 3.6 (3.3 - 3.8) | 5.8 (5.5 - 6.2) | 7 (6.5 - 7.4) | 4.6 (3.9 - 5.2) |
| 2003 | 2.3 (2.2 - 2.5) | 3.6 (3.4 - 3.9) | 5.9 (5.5 - 6.2) | 7.2 (6.8 - 7.7) | 5.3 (4.6 - 6) |
| 2004 | 2.2 (2.1 - 2.3) | 3.3 (3.1 - 3.5) | 5.8 (5.4 - 6.1) | 6.7 (6.3 - 7.1) | 5.5 (4.9 - 6.2) |
| 2005 | 2.2 (2.1 - 2.4) | 3.6 (3.4 - 3.8) | 5.8 (5.4 - 6.1) | 7.2 (6.7 - 7.6) | 5.3 (4.6 - 5.9) |
| 2006 | 2.2 (2 - 2.3) | 3.7 (3.4 - 3.9) | 5.5 (5.1 - 5.8) | 7.4 (6.9 - 7.9) | 4.8 (4.2 - 5.4) |
| 2007 | 1.9 (1.8 - 2.1) | 3.5 (3.3 - 3.7) | 5.4 (5.1 - 5.8) | 7.1 (6.6 - 7.5) | 5.3 (4.7 - 6) |
| 2008 | 1.9 (1.8 - 2) | 3.8 (3.6 - 4) | 5.5 (5.1 - 5.8) | 6.7 (6.3 - 7.2) | 5.6 (4.9 - 6.2) |
| 2009 | 1.8 (1.7 - 1.9) | 3.7 (3.5 - 3.9) | 5.3 (5 - 5.6) | 6.8 (6.3 - 7.2) | 4.6 (4 - 5.2) |
| 2010 | 1.8 (1.7 - 1.9) | 4 (3.8 - 4.2) | 5.3 (5 - 5.6) | 7.2 (6.7 - 7.6) | 4.8 (4.3 - 5.4) |
| 2011 | 2 (1.9 - 2.1) | 4.5 (4.3 - 4.7) | 6.2 (5.9 - 6.6) | 8.6 (8.1 - 9.1) | 6.8 (6.2 - 7.5) |
| 2012 | 2 (1.9 - 2.1) | 4.7 (4.5 - 5) | 6.5 (6.2 - 6.9) | 8.8 (8.3 - 9.3) | 7.3 (6.6 - 7.9) |
| 2013 | 1.8 (1.6 - 1.9) | 4.1 (3.9 - 4.3) | 5.5 (5.2 - 5.8) | 6.9 (6.5 - 7.4) | 6.2 (5.5 - 6.8) |
| 2014 | 1.8 (1.7 - 1.9) | 4.2 (4 - 4.4) | 5.6 (5.3 - 5.9) | 7 (6.5 - 7.4) | 5.7 (5.1 - 6.3) |
| 2015 | 1.8 (1.7 - 2) | 4.2 (4 - 4.4) | 5.8 (5.6 - 6.1) | 7.7 (7.2 - 8.1) | 6.2 (5.5 - 6.8) |
| 2016 | 1.6 (1.4 - 1.7) | 4.3 (4.1 - 4.5) | 5.9 (5.7 - 6.2) | 7.7 (7.3 - 8.2) | 6.5 (5.9 - 7.2) |
| 2017 | 1.6 (1.5 - 1.7) | 4.2 (4 - 4.4) | 6.4 (6.1 - 6.7) | 7.8 (7.3 - 8.3) | 6.7 (6.1 - 7.3) |
| 2018 | 1.7 (1.6 - 1.8) | 4.1 (3.9 - 4.3) | 6.5 (6.2 - 6.7) | 8.4 (7.9 - 8.8) | 7.1 (6.4 - 7.7) |
| 2019 | 1.7 (1.6 - 1.9) | 4.2 (4 - 4.4) | 6.8 (6.5 - 7.1) | 8.8 (8.3 - 9.2) | 7.1 (6.5 - 7.7) |
| 2020 | 1.9 (1.8 - 2) | 4.5 (4.3 - 4.7) | 7.3 (7 - 7.6) | 9.6 (9.1 - 10.1) | 8.5 (7.8 - 9.2) |
| 2021 | 2.4 (2.2 - 2.5) | 5.3 (5.1 - 5.5) | 9 (8.7 - 9.4) | 11.5 (11 - 12) | 10.6 (9.8 - 11.4) |
| 2022 | 2.5 (2.4 - 2.7) | 5.9 (5.7 - 6.2) | 10.3 (10 - 10.7) | 12.5 (11.9 - 13) | 11.1 (10.3 - 11.9) |
| 2023 | 2.4 (2.3 - 2.6) | 5.5 (5.3 - 5.7) | 9.7 (9.4 - 10.1) | 12.7 (12.2 - 13.2) | 11.9 (11.1 - 12.8) |
| **Total** | 2.0 (1.9 - 2.1) | 4.1 (4.0 - 4.3) | 6.3 (6.0 - 6.7) | 8.0 (7.5 - 8.5) | 6.5 (5.8 - 7.1) |

**Supplemental Table 6.** Age group stratified Kidney Failure and Liver Cirrhosis-related crude mortality rates per 100,000 among adults aged 45 and above in the United States, 1999 to 2023.

| **Age-Adjusted Rate /100,000 (95% CI)** | | |
| --- | --- | --- |
| **Year** | **Metropolitan** | **Non-Metropolitan** |
| 1999 | 3.7 (3.6 - 3.8) | 3.4 (3.1 - 3.7) |
| 2000 | 3.9 (3.8 - 4.1) | 3.4 (3.1 - 3.6) |
| 2001 | 3.9 (3.8 - 4.1) | 3.5 (3.2 - 3.7) |
| 2002 | 4.1 (3.9 - 4.2) | 3.7 (3.4 - 4) |
| 2003 | 4.2 (4 - 4.3) | 3.9 (3.6 - 4.2) |
| 2004 | 3.9 (3.8 - 4.1) | 3.5 (3.2 - 3.8) |
| 2005 | 4 (3.9 - 4.1) | 3.9 (3.6 - 4.2) |
| 2006 | 4 (3.9 - 4.2) | 3.5 (3.2 - 3.8) |
| 2007 | 3.9 (3.8 - 4) | 3.3 (3.1 - 3.6) |
| 2008 | 3.8 (3.7 - 4) | 3.7 (3.4 - 3.9) |
| 2009 | 3.7 (3.6 - 3.8) | 3.7 (3.5 - 4) |
| 2010 | 3.8 (3.7 - 3.9) | 4 (3.7 - 4.3) |
| 2011 | 4.5 (4.3 - 4.6) | 4.6 (4.3 - 4.9) |
| 2012 | 4.7 (4.5 - 4.8) | 4.5 (4.2 - 4.8) |
| 2013 | 3.9 (3.8 - 4.1) | 4 (3.7 - 4.2) |
| 2014 | 3.9 (3.8 - 4) | 4.2 (3.9 - 4.5) |
| 2015 | 4.2 (4 - 4.3) | 4.1 (3.8 - 4.4) |
| 2016 | 4 (3.9 - 4.2) | 4.3 (4 - 4.6) |
| 2017 | 4.1 (4 - 4.2) | 4.5 (4.2 - 4.7) |
| 2018 | 4.2 (4.1 - 4.4) | 4.7 (4.4 - 5) |
| 2019 | 4.3 (4.2 - 4.4) | 5.1 (4.8 - 5.4) |
| 2020 | 4.7 (4.6 - 4.8) | 5.7 (5.4 - 6) |
| **Total** | 4.1 (4.1 - 4.1) | 4.1 (4 - 4.2) |

**Supplemental Table 7.** Urbanization stratified Kidney Failure and Liver Cirrhosis-related age-adjusted mortality rates per 100,000 among adults aged 45 and above in the United States, 1999 to 2020.

| **Age-Adjusted Rate /100,000 (95% CI)** | | | | |
| --- | --- | --- | --- | --- |
| **Year** | **Northeast** | **Midwest** | **South** | **West** |
| 1999 | 3.5 (3.2 - 3.8) | 3.3 (3.1 - 3.6) | 3.6 (3.4 - 3.8) | 4 (3.8 - 4.3) |
| 2000 | 3.9 (3.6 - 4.2) | 3.5 (3.3 - 3.8) | 4 (3.8 - 4.2) | 3.8 (3.5 - 4.1) |
| 2001 | 3.8 (3.5 - 4.1) | 3.6 (3.3 - 3.8) | 3.9 (3.7 - 4.1) | 4.2 (3.9 - 4.5) |
| 2002 | 3.7 (3.4 - 3.9) | 3.8 (3.6 - 4.1) | 4.2 (4 - 4.4) | 4.2 (3.9 - 4.4) |
| 2003 | 4 (3.8 - 4.3) | 3.7 (3.5 - 4) | 4.2 (4 - 4.4) | 4.3 (4 - 4.6) |
| 2004 | 3.6 (3.4 - 3.9) | 3.6 (3.3 - 3.8) | 4 (3.8 - 4.2) | 4.1 (3.8 - 4.4) |
| 2005 | 3.7 (3.4 - 3.9) | 3.6 (3.4 - 3.8) | 4.4 (4.2 - 4.6) | 4.1 (3.8 - 4.3) |
| 2006 | 3.8 (3.5 - 4.1) | 3.8 (3.6 - 4) | 4.1 (3.9 - 4.3) | 3.9 (3.7 - 4.2) |
| 2007 | 3.4 (3.2 - 3.7) | 3.5 (3.3 - 3.7) | 4.2 (4 - 4.4) | 3.7 (3.5 - 4) |
| 2008 | 3.4 (3.2 - 3.7) | 3.5 (3.2 - 3.7) | 4.3 (4.1 - 4.5) | 3.7 (3.5 - 3.9) |
| 2009 | 3.2 (2.9 - 3.4) | 3.3 (3.1 - 3.5) | 4.2 (4 - 4.4) | 3.7 (3.5 - 3.9) |
| 2010 | 3.5 (3.3 - 3.8) | 3.4 (3.1 - 3.6) | 4.3 (4.1 - 4.5) | 3.9 (3.6 - 4.1) |
| 2011 | 4 (3.7 - 4.3) | 4.1 (3.8 - 4.3) | 5 (4.8 - 5.2) | 4.5 (4.2 - 4.7) |
| 2012 | 3.7 (3.4 - 3.9) | 4 (3.8 - 4.2) | 5.4 (5.1 - 5.6) | 5 (4.7 - 5.2) |
| 2013 | 3.2 (3 - 3.5) | 3.6 (3.3 - 3.8) | 4.6 (4.4 - 4.8) | 3.8 (3.6 - 4) |
| 2014 | 3.4 (3.2 - 3.6) | 3.5 (3.3 - 3.7) | 4.6 (4.4 - 4.8) | 3.9 (3.6 - 4.1) |
| 2015 | 3.4 (3.2 - 3.6) | 3.5 (3.3 - 3.8) | 4.8 (4.6 - 5) | 4.2 (4 - 4.5) |
| 2016 | 3.3 (3.1 - 3.5) | 3.7 (3.5 - 3.9) | 4.7 (4.5 - 4.9) | 4.1 (3.9 - 4.4) |
| 2017 | 3.1 (2.9 - 3.3) | 3.9 (3.7 - 4.1) | 4.8 (4.6 - 5) | 4.4 (4.1 - 4.6) |
| 2018 | 3.2 (3 - 3.4) | 3.8 (3.6 - 4) | 5.2 (5 - 5.4) | 4.2 (4 - 4.4) |
| 2019 | 3.6 (3.4 - 3.8) | 4.1 (3.9 - 4.3) | 5.2 (5 - 5.4) | 4.2 (4 - 4.4) |
| 2020 | 3.9 (3.7 - 4.1) | 4.5 (4.3 - 4.7) | 5.6 (5.4 - 5.8) | 4.9 (4.6 - 5.1) |
| 2021 | 4.6 (4.4 - 4.9) | 5.4 (5.1 - 5.7) | 6.8 (6.6 - 7) | 6 (5.8 - 6.3) |
| 2022 | 5.2 (4.9 - 5.4) | 6.1 (5.8 - 6.4) | 7.2 (7 - 7.4) | 6.8 (6.5 - 7.1) |
| 2023 | 4.7 (4.4 - 4.9) | 6.1 (5.8 - 6.3) | 7.2 (7 - 7.4) | 6.5 (6.2 - 6.7) |
| **Total** | 3.7 (3.5 - 4) | 4 (3.7 - 4.2) | 4.8 (4.6 - 5) | 4.4 (4.1 - 4.6) |

**Supplemental Table 8.** Region-stratified Kidney Failure and Liver Cirrhosis-related age-adjusted mortality rates per 100,000 among adults aged 45 and above in the United States, 1999 to 2023.

| **State** | **Age-Adjusted Rate /100,000 (95% CI)** | **Percentile** |
| --- | --- | --- |
| Wyoming | 2.2 (1.7 - 2.6) | 0% |
| Nebraska | 2.5 (2.3 - 2.8) | 2% |
| Iowa | 2.6 (2.4 - 2.7) | 4% |
| Louisiana | 2.6 (2.5 - 2.8) | 4% |
| Montana | 2.7 (2.4 - 3) | 8% |
| New York | 2.7 (2.6 - 2.8) | 8% |
| Idaho | 2.8 (2.5 - 3.1) | 12% |
| Utah | 2.8 (2.5 - 3) | 12% |
| Alaska | 2.9 (2.3 - 3.4) | 16% |
| Kansas | 2.9 (2.7 - 3.1) | 16% |
| Colorado | 3 (2.8 - 3.2) | 20% |
| Florida | 3 (3 - 3.1) | 20% |
| Georgia | 3 (2.8 - 3.1) | 20% |
| Maine | 3.1 (2.8 - 3.4) | 26% |
| Oregon | 3.1 (2.9 - 3.3) | 26% |
| North Dakota | 3.2 (2.7 - 3.6) | 30% |
| Vermont | 3.2 (2.7 - 3.7) | 30% |
| Wisconsin | 3.2 (3 - 3.3) | 30% |
| Nevada | 3.3 (3.1 - 3.5) | 36% |
| South Dakota | 3.3 (2.9 - 3.8) | 36% |
| Missouri | 3.4 (3.2 - 3.5) | 40% |
| Arizona | 3.5 (3.3 - 3.6) | 42% |
| Illinois | 3.6 (3.5 - 3.7) | 44% |
| Minnesota | 3.6 (3.4 - 3.7) | 44% |
| New Jersey | 3.7 (3.5 - 3.8) | 48% |
| Michigan | 3.8 (3.7 - 3.9) | 50% |
| New Hampshire | 3.9 (3.6 - 4.3) | 52% |
| Virginia | 3.9 (3.7 - 4) | 52% |
| Washington | 3.9 (3.7 - 4.1) | 52% |
| Arkansas | 4 (3.7 - 4.2) | 58% |
| Connecticut | 4 (3.8 - 4.2) | 58% |
| Hawaii | 4 (3.6 - 4.4) | 58% |
| Maryland | 4 (3.9 - 4.2) | 58% |
| Oklahoma | 4 (3.8 - 4.2) | 58% |
| Mississippi | 4.1 (3.9 - 4.4) | 68% |
| Massachusetts | 4.2 (4 - 4.3) | 70% |
| Pennsylvania | 4.2 (4.1 - 4.3) | 70% |
| North Carolina | 4.4 (4.3 - 4.6) | 74% |
| Ohio | 4.4 (4.2 - 4.5) | 74% |
| Alabama | 4.6 (4.4 - 4.8) | 78% |
| Delaware | 4.7 (4.2 - 5.1) | 80% |
| South Carolina | 4.8 (4.6 - 5) | 82% |
| California | 4.9 (4.8 - 5) | 84% |
| Indiana | 4.9 (4.7 - 5.1) | 84% |
| District of Columbia | 5 (4.4 - 5.7) | 88% |
| Tennessee | 5 (4.8 - 5.1) | 88% |
| New Mexico | 5.2 (4.8 - 5.5) | 92% |
| Kentucky | 5.3 (5.1 - 5.5) | 94% |
| Rhode Island | 5.4 (4.9 - 5.9) | 96% |
| West Virginia | 5.9 (5.5 - 6.2) | 98% |
| Texas | 7.6 (7.4 - 7.7) | 100% |

**Supplemental Table 9.** State-stratified Kidney Failure and Liver Cirrhosis-related age-adjusted mortality rates per 100,000 and their respective percentiles among adults aged 45 and above in the United States, 1999 to 2020.

**Supplementary Figures**


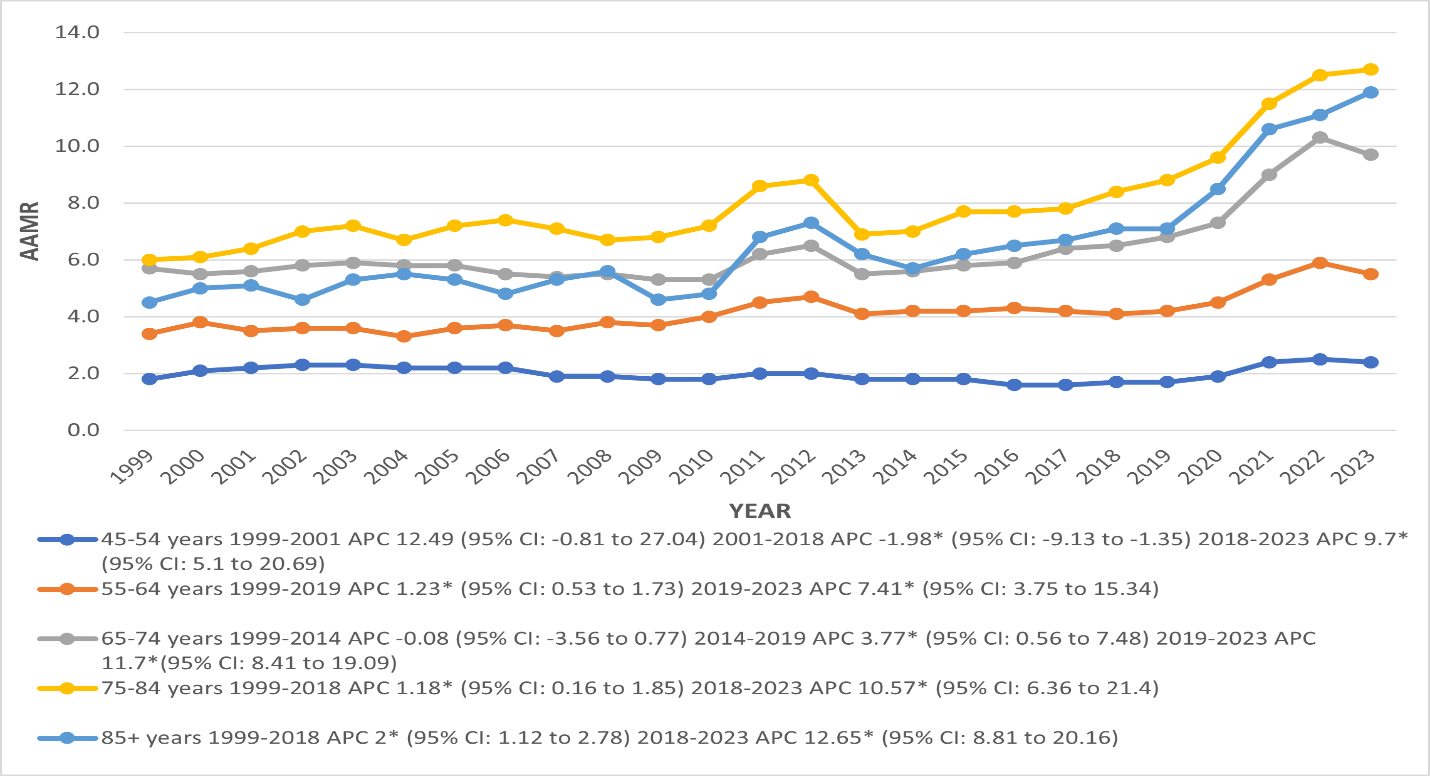


**Supplementary Figure 1.** Trends in Kidney Failure and Liver Cirrhosis-related age-adjusted mortality rates per 100,000, stratified by age group among adults aged 45 and above in the United States, 1999 to 2023.

APC = Annual Percentage Change, CI = Confidence Interval.

*Indicates that the Annual Percentage Change (APC) is significantly different from zero at α = 0.05.


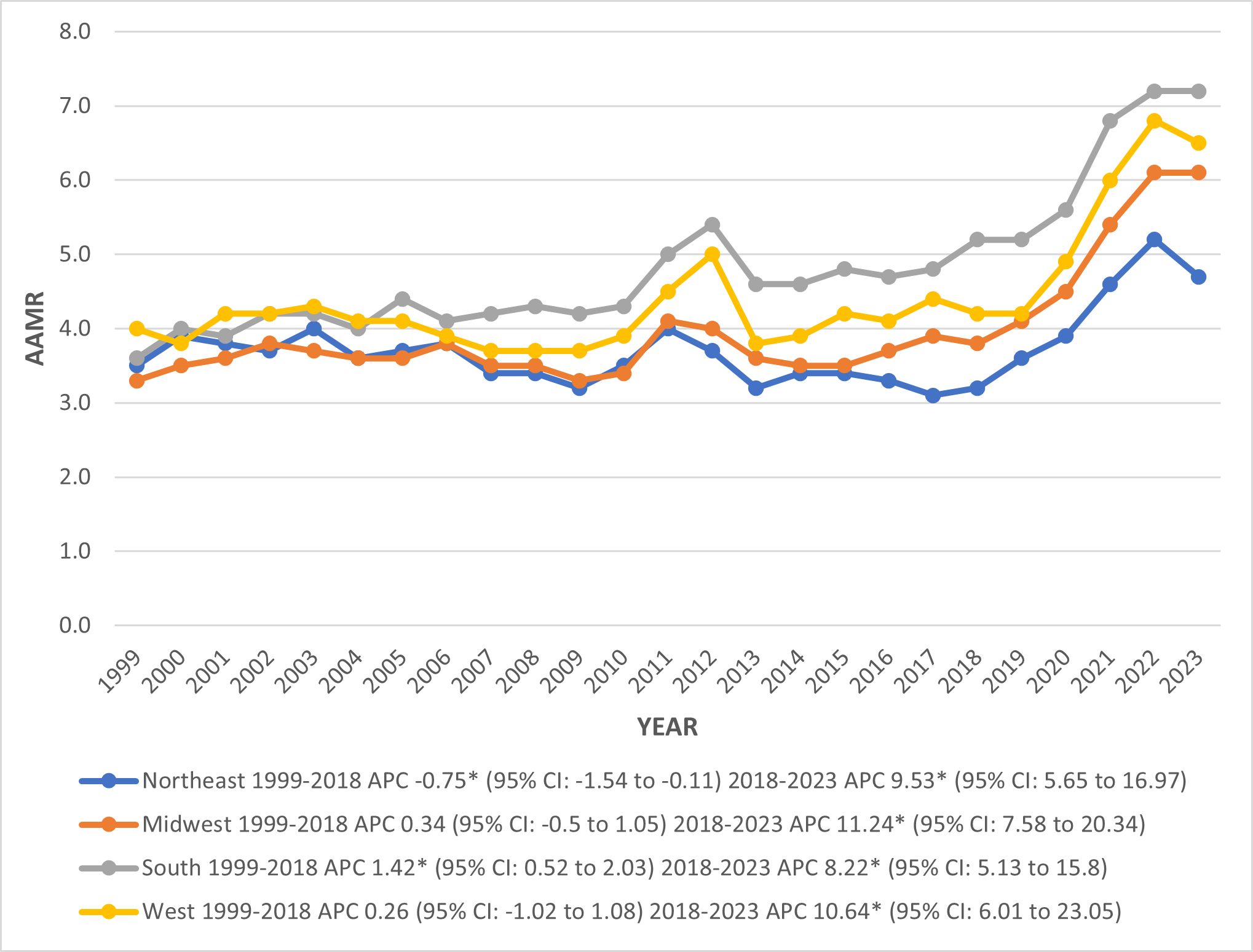


**Supplementary Figure 2.** Trends in Kidney Failure and Liver Cirrhosis-related age-adjusted mortality rates per 100,000, stratified by census region among adults aged 45 and above in the United States, 1999 to 2023.

APC = Annual Percentage Change, CI = Confidence Interval.

*Indicates that the Annual Percentage Change (APC) is significantly different from zero at α = 0.05.


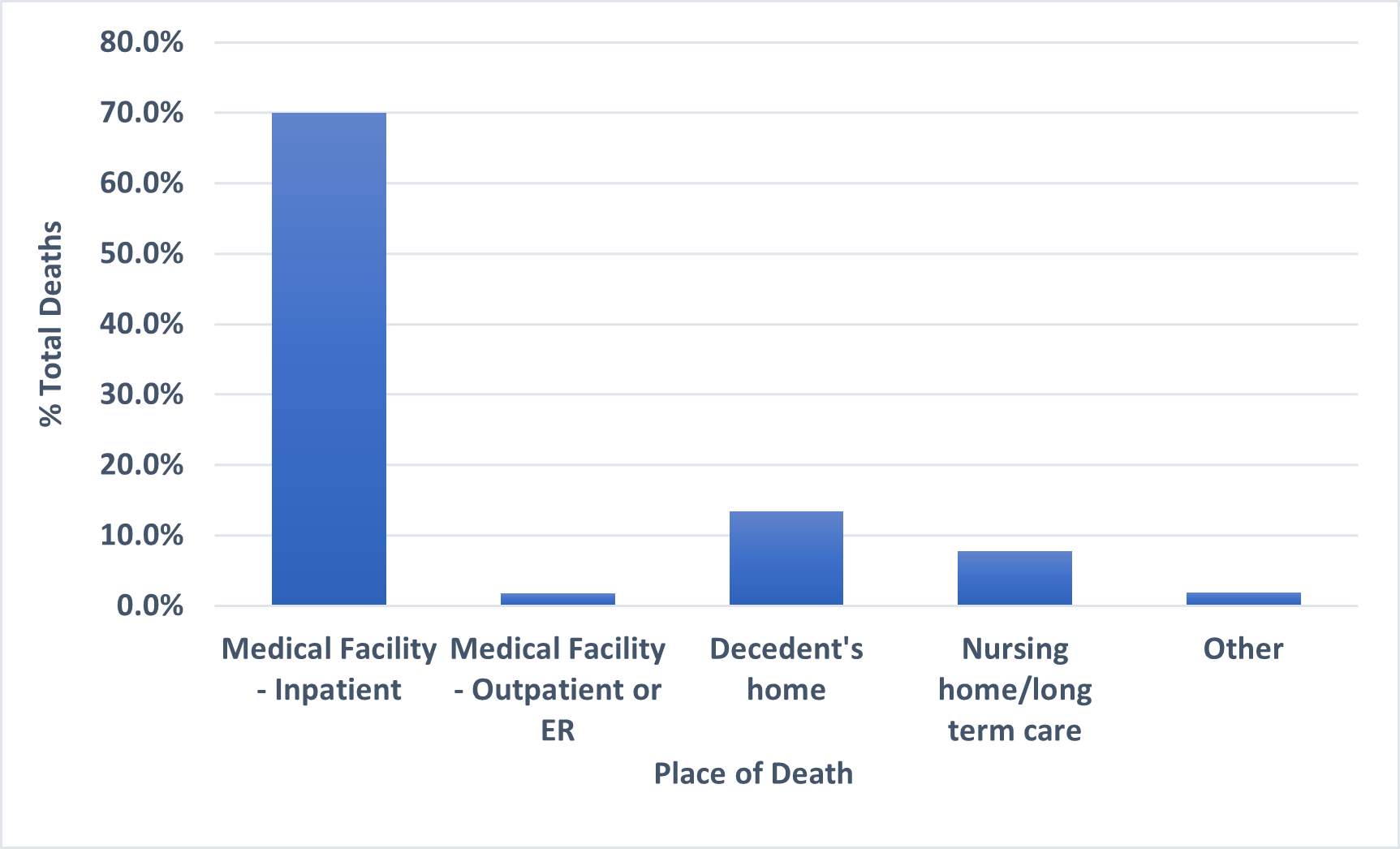


**Supplementary Figure 3.** Percent total deaths of Kidney Failure associated with Liver Cirrhosis by place of deaths among adults aged 45 and above in the United States, 1999 to 2023.


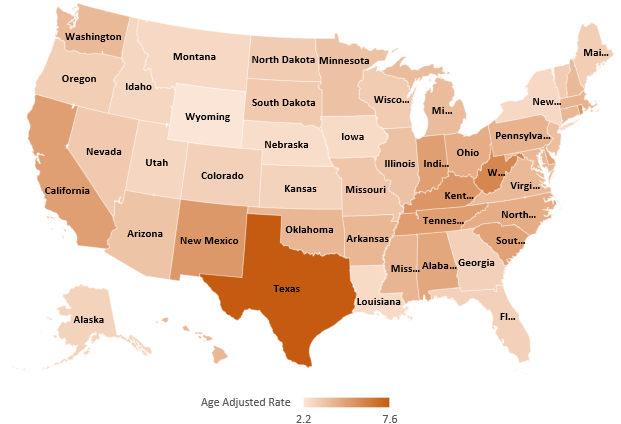


**Supplementary Figure 4.** Kidney Failure and Liver Cirrhosis-related age-adjusted mortality rates per 100,000, stratified by states among adults aged 45 and above in the United States, 1999 to 2020.
